# Predicting virologically confirmed influenza using school absences in Allegheny County, Pennsylvania, USA during the 2007‐2015 influenza seasons

**DOI:** 10.1111/irv.12865

**Published:** 2021-09-03

**Authors:** Talia M. Quandelacy, Shanta Zimmer, Justin Lessler, Charles Vukotich, Rachel Bieltz, Kyra H. Grantz, David Galloway, Jonathan M. Read, Yenlik Zheteyeva, Hongjiang Gao, Amra Uzicanin, Derek A. T. Cummings

**Affiliations:** ^1^ Johns Hopkins University Baltimore MD USA; ^2^ University of Colorado Anschutz Medical Campus Aurora CO USA; ^3^ University of Pittsburgh Pittsburgh PA USA; ^4^ University of Colorado Denver CO USA; ^5^ University of Florida Gainesville FL USA; ^6^ Lancaster University Lancaster UK; ^7^ Centers for Disease Control and Prevention Atlanta GA USA

**Keywords:** human influenza, prediction, school‐aged children, surveillance

## Abstract

**Background:**

Children are important in community‐level influenza transmission. School‐based monitoring may inform influenza surveillance.

**Methods:**

We used reported weekly confirmed influenza in Allegheny County during the 2007 and 2010‐2015 influenza seasons using Pennsylvania's Allegheny County Health Department all‐age influenza cases from health facilities, and all‐cause and influenza‐like illness (ILI)‐specific absences from nine county school districts. Negative binomial regression predicted influenza cases using all‐cause and illness‐specific absence rates, calendar week, average weekly temperature, and relative humidity, using four cross‐validations.

**Results:**

School districts reported 2 184 220 all‐cause absences (2010‐2015). Three one‐season studies reported 19 577 all‐cause and 3012 ILI‐related absences (2007, 2012, 2015). Over seven seasons, 11 946 confirmed influenza cases were reported. Absences improved seasonal model fits and predictions. Multivariate models using elementary school absences outperformed middle and high school models (relative mean absolute error (relMAE) = 0.94, 0.98, 0.99). K‐5 grade‐specific absence models had lowest mean absolute errors (MAE) in cross‐validations. ILI‐specific absences performed marginally better than all‐cause absences in two years, adjusting for other covariates, but markedly worse one year.

**Conclusions:**

Our findings suggest seasonal models including K‐5th grade absences predict all‐age‐confirmed influenza and may serve as a useful surveillance tool.

## 1 INTRODUCTION

Influenza surveillance utilizes multiple data sources, including syndromic indicators, laboratory‐confirmed cases, and deaths.^1^ Non‐clinical sources have the potential to complement clinical and laboratory data and improve influenza prediction efforts.^2^ Student absenteeism is a real‐time school‐based indicator for influenza surveillance tool. It is advantageous for being widely available in real time, having minimal reporting delays, and being relatively low cost and is a reasonable proxy for influenza infections since school‐age children (5‐ to 17‐year‐olds) experience higher infections compared to other age groups^3^ and contribute to household and community‐level transmission.^4^

Previous studies used school‐based surveillance (ie, absence duration^5^ or causes)^6^, ^7^ to identify patterns correlated with influenza‐ or ILI‐related cases, primarily at a city‐level, but usefulness of school absenteeism as a surveillance indicator in these studies has been mixed. Using ILI‐specific absence duration predicted 2005‐2008 outbreaks well in Japan with high sensitivity and specificity,^5^ but a similar approach using city‐level all‐cause absences from 2005 to 2009 had low predictive ability when predicting outbreaks in New York City.^6^ Absence patterns correlated well with sentinel surveillance in Hong Kong showing similar peaks in absenteeism and ILI consultation and influenza detection rates, but ILI‐specific absences had low specificity.^8^ The varied conclusions of these studies could be from differing school‐ and absence‐type captured, and short surveillance periods, but other types of absence data could have utility.

Grade‐specific differences in absences have not been explored as a predictor of influenza but may correlate better to high‐risk infections‐groups. Given the variation of infection burden and proportion of illness‐related absences by age, particular individual school levels and grades may serve as a proxy for these high‐risk infection groups. School absenteeism may also be useful for detecting underlying viral changes in transmission. Unusual patterns of school absences arising across different periods of time have also been correlated to detecting changes in influenza A and B viruses^9^ and have been attributed to detecting the re‐emergence of an influenza B/Victoria antigenic group.^10^ The varied study findings of school absenteeism suggest further assessment is needed.

Here, we evaluated how school absences models predicted weekly confirmed influenza cases in Allegheny County, Pennsylvania over multiple influenza seasons. We compared predictions from all‐cause absence models for the 2010‐2015 influenza seasons at varying administrative levels. We also compared predictions for individual influenza seasons (2007‐2008, 2012‐2013, and 2015‐2016) from models including all‐cause and ILI‐related absences from three school‐based cohort studies.

## 2 METHODS

### 2.1 Ethics

Our analyses used only de‐identified data. We obtained Institutional Research Board approval from University of Pittsburgh (PRO13100580), Johns Hopkins Bloomberg School of Public Health (IRB #5474), Centers for Disease Control and Prevention (IRB#00000319), and the Allegheny County Department of Health.

### 2.2 Data

Allegheny County Health Department (ACHD) provided virologically confirmed influenza case data (N = 11 946) for 2007‐2008, and 2010‐2015 influenza seasons. A reported confirmed influenza case was a positive laboratory‐confirmed test (ie, rapid diagnostic test, reverse‐transcriptase polymerase chain reaction, or viral culture) reported by hospital emergency departments or sentinel medical providers in Allegheny Country from any individual experiencing influenza‐like illness. Weekly all‐cause absences and school enrollment data for 2010‐2015 came from nine Allegheny County school districts. Six districts provided grade‐specific absences (Supplemental Text and Supplemental Table 1). Grade level served as an age‐proxy, since student demographics (ie, age, gender, or vaccination status) were unavailable. Additional all‐cause and cause‐specific absences came from three school‐based cohort studies (Pittsburgh Influenza Prevention Project (PIPP) during the 2007‐2008 season (10 K‐5 schools), Social Mixing and Respiratory Transmission in School study (SMART) during the 2012‐2013 season (eight K‐12 schools), and Surveillance Monitoring of Absences and Respiratory Transmission (SMART^2^) study during the 2015‐2016 season (nine K‐12 schools)). Cohort studies used similar absence collection protocols.^11^

Greater Pittsburgh area daily minimum and maximum temperature and relative humidity data came from the National Oceanographic and Atmospheric Administration's National Climatic Data Center.^12^ We used temperature and relative humidity (a proxy for absolute humidity) given their effects on influenza transmission (ie, viral dispersal and survival).^13^, ^14^ Allegheny County population data came from US Census Bureau's yearly estimates for 2007, 2008, and 2010‐2015.^15^

Our primary outcome was weekly confirmed influenza infections reported in Allegheny County during 2007‐2008 and 2010‐2015 seasons. Influenza infection was defined as any virologically confirmed case reported by a health provider in Allegheny County during CDC‐defined influenza seasons (ie, 40th calendar week to the 20th calendar week of the subsequent year).^16^ Weekly influenza cases were the total cases reported each week, excluding cases occurring during school closures (eg, spring break, federal holidays, weekends).

All‐cause absences were defined as a full or partial school day missed for any reason. Cause‐specific absences were a full or partial school day missed due to influenza‐like illness (ie, fever (>37C) and either cough, sore throat, runny nose, or congestion). We restricted school absences to periods overlapping the influenza seasons to examine absence patterns during influenza circulation, and excluded weekends, observed federal holidays, and school breaks. Weekly school absences were the total absences reported in one school week (ie, if no observed holidays, five days in a school week). Weekly absence rates were total absences in a week, divided by the total students enrolled times the number of school days in a given week.

We used the daily minimum and maximum temperatures to obtain the average daily temperature. Average daily temperature and relative humidity were each aggregated to the week level to obtain the weekly average temperature and relative humidity for each influenza season.

### 2.3 Statistical analysis

We predicted weekly influenza cases over seven influenza seasons using negative binomial regression models. Continuous predictor variables were weekly absence rates (lagged by one‐week), calendar week, average weekly temperature, and relative humidity. Models used predictors individually and in combination. The offset term represented the estimated annual Allegheny Country population for 2007 and 2010‐2015 influenza seasons. Seasonal variables (calendar week, temperature, and relative humidity) accounted for temporal and climatic variation of influenza. We modeled calendar week to account for seasonal trends, average weekly temperature, and average weekly relative humidity as nonlinear terms using thin‐plate penalized splines in generalized additive models (mgcv R package).^17^ Models including school‐ (ie, elementary, middle and high school) and grade‐specific absences (alone and in combination) were evaluated to determine whether finer administrative‐level absences improved model fits and predictions. From three school‐based cohort studies, we compared all‐cause and cause‐specific absence model performance for single seasons (2007, 2012, and 2015), and pooled over these seasons.

Sensitivity analyses examined absence duration, and lagged influenza, and kindergarten‐specific absences. We used one‐day and two‐day or longer absences to assess the impact of absence duration on weekly influenza predictions from 2010 to 2015. Models used one‐day absences, and absences two days or longer individually, together, and in models containing average temperature, relative humidity, and calendar week. We also assessed weekly influenza predictions from models including one‐week‐lagged influenza cases, and county‐level and kindergarten‐specific all‐cause absences.

We compared nested and non‐nested models using Akaike's Information Criterion corrected for small sample sizes (AICc). Decreased AICc signified improved model fits. Two‐sided 5% alpha‐level determined statistical significance. Analyses used R version 3.1.3 (R Foundation for Statistical Computing, 2016).

### 2.4 Model validation and predictions

We validated our models using training (ie, in‐sample) and testing (ie, out‐of‐sample) data generated from the following four (independent) sampling approaches: (i) randomly sampled 80% of weeks without replacement; (ii) leave out 52 non‐contiguous randomly sampled weeks; (iii) leave out 20% of randomly sampled schools, and (iv) leave one influenza season out (ie, model training used all but one season and the out‐of‐sample season was used for model testing) to account for influenzas’ seasonal variation. Estimated *R* ^2^ used linear regressions of out‐of‐sample observed influenza cases (outcome) and predicted cases (independent variable). Prediction metrics used mean absolute error (MAE) and relative mean absolute error (relMAE). Mean absolute error was defined as the mean of the absolute value of model prediction errors.^18^ Relative MAE is the ratio comparing a model's MAE to a reference MAE (ie, from a model including calendar week, and average weekly temperature, and relative humidity), where relMAE of 1.0 indicated the same prediction error for two models. We visually compared observed and predicted cumulative distributions and time‐series of influenza cases.

## 3 RESULTS

### 3.1 Characteristics of influenza and school absences

**FIGURE 1:**
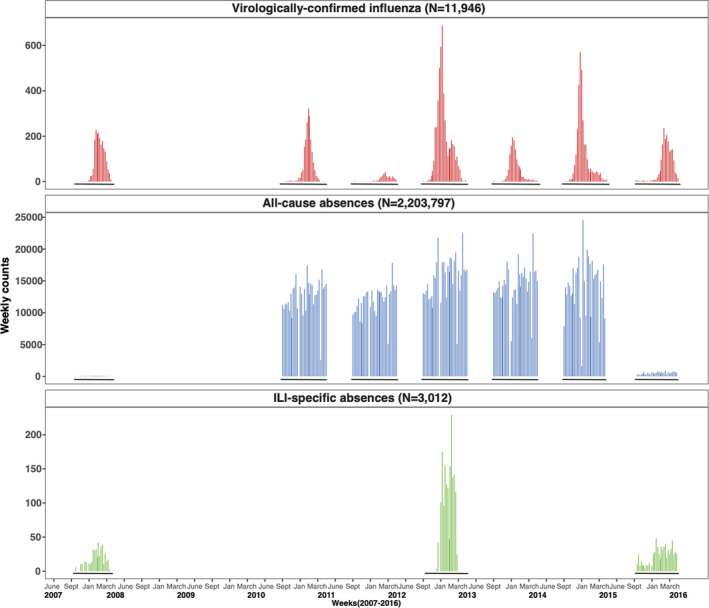
Weekly reported virologically confirmed influenza cases, and all‐cause and influenza‐like‐illness (ILI) specific absences in Allegheny County, Pennsylvania, USA, during influenza seasons from 2007 to 2015. Surveillance of influenza cases during each influenza season in Allegheny County occurred from the 40th week of one year to the 20th week of the following year (solid black lines). All‐cause absences were collected for the entire school year for each school district, and data were restricted to their respective influenza seasons. Nine school districts within Allegheny contributed to weekly counts of all‐cause absences. Additionally, all‐cause and ILI‐specific absences were collected during independent influenza seasons for three separate studies: 2007‐2008 (PIPP study), 2012‐2013 (SMART study), and 2015‐2016 (SMART^2^ study). Absence surveillance data were not collected during the 2008‐2009 or 2009‐2010 influenza seasons. White space on the x‐axis reflects periods when data were not collected for this analysis, whereas black lines on the x‐axis (in negative y values) indicate time periods when data are available. The Allegheny County student population averaged 43,636 students across the nine school districts, comprising 122 schools (57 elementary, 20 middle, and 18 high schools, and 24 charter/independent schools). County‐level data for 2010‐2011 season were not available for 3 school districts

Over seven influenza seasons, 11 946 confirmed influenza cases were reported to ACHD (Supplemental Table 1). Influenza type A predominated most seasons, similar to national patterns.^19^, ^20^, ^21^, ^22^, ^23^, ^24^ Overall, 9350 type A (1397 A/H3N2 and 1115 A/H1N1 subtypes) cases, 2453 type B cases, and 143 un‐typed cases were reported. The 2011‐2012 and 2014‐2015 seasons were the lowest (301 cases) and highest (3150 cases) transmission seasons in Allegheny Country, like national trends. Within seasons, cases peaked in the winter whereas county‐level absences varied throughout the year (Figure 1).

During the 2010‐2015 seasons, county school districts reported 2 184 200 total absences (Figure 1), averaging 6.5 weekly absences/100 students (interquartile range [IQR: 5.6, 7.7]) (Supplemental Table 1). High schools had the highest average absence rates (9.4 weekly absences/100 students, IQR: 8.1,10.8), followed by middle schools (6.3 weekly absences/100 students, IQR: 5.4, 7.7) and elementary schools (5.3 weekly absences/100 students, IQR: 4.0, 6.6). Study schools reported 20,128 all‐cause and 3,012 ILI‐specific absences among 11,660 students (Supplemental Table 1). The SMART^2^ study had the highest average weekly all‐cause absence rates (2.2 weekly absences/100 students (IQR: 1.8, 2.5)), while the SMART study had the highest ILI‐specific absence rates (1.1 weekly absences/100 students (IQR: 0.7, 1.4)).

### 3.2 Influenza predictions using county‐level absences

We evaluated negative binomial models of seasonal variables (ie, calendar week, average weekly temperature, and relative humidity) alone, and including weekly all‐cause county‐level school absences at one‐, two‐, and three‐week lags. One‐ and third‐week‐lagged absences had similar model performance (Supplemental Table 2); therefore, we used one‐week‐lagged absences in all models to better reflect influenza's infectious period (ie, 1‐week spread).^25^ Compared to seasonal models, AICs of in‐sample models including calendar week, average weekly temperature, average weekly relative humidity, and one‐week‐lagged weekly county‐level all‐cause absences either stayed the same or slightly worsened (**∆**AICc = 2, 1, and 0, Table 1), whereas models of calendar week, average weekly temperature, and one‐week‐lagged weekly absences had slightly improved fits (**∆**AICc = −4, −4, −4, Table 1). For prediction performance, MAEs either stayed the same or decreased when including one‐week‐lagged weekly absences in models of calendar week, average weekly temperature, and relative humidity relative to seasonal‐only models (relMAE = 0.95, 1.0, & 0.95, Table 1).

**TABLE 1:** Fit and Performance of negative binomial models of seasonal variables including and excluding one‐week‐lagged county‐level all‐cause school absence rates to predict weekly confirmed influenza cases in Allegheny County, Pennsylvania during the 2010‐2015 seasons

Model validation		Leave 20% randomly sampled out data (n = 124)			Leave 52 randomly sampled weeks out (n = 106)			Leave 20% randomly sampled schools out (n = 159)		
Model^a^	Variables	*df*	∆AICc^b^	Relative MAE^b^	*df*	∆AICc^b^	Relative MAE^b^	*df*	∆AICc^b^	Relative MAE^b^
1 (Ref.)	Week, temperature, RH	8.5	0.0	1.0	8.8	0.0	1.0	10.0	0.0	1.0
2	Week, temperature, all‐cause absence rates	7.3	−4.0	0.97	7.2	−4.0	1.05	7.4	−4.0	1.0
3	Week, RH, all‐cause absence rates	7.9	−1.0	1.23	8.0	−1.0	1.2	9.5	−1.0	1.27
4	Week, temperature, RH, all‐cause absence rates	8.8	2.0	0.95	8.9	1.0	1.0	10.3	0.0	0.95

Abbreviations: ∆AICc, change in Akaike's Information Criterion corrected for small sample size; RH, relative humidity.

^a^ Each model used negative binomial regression and used generalized additive models to estimate degrees of freedom for nonlinear (ie, spline) variables.

^b^ Changes in AICc and relMAE compared all models to the reference (model 1), a seasonal variables‐only model that contains calendar week, average weekly temperature, and average weekly relative humidity.

**FIGURE 2:**
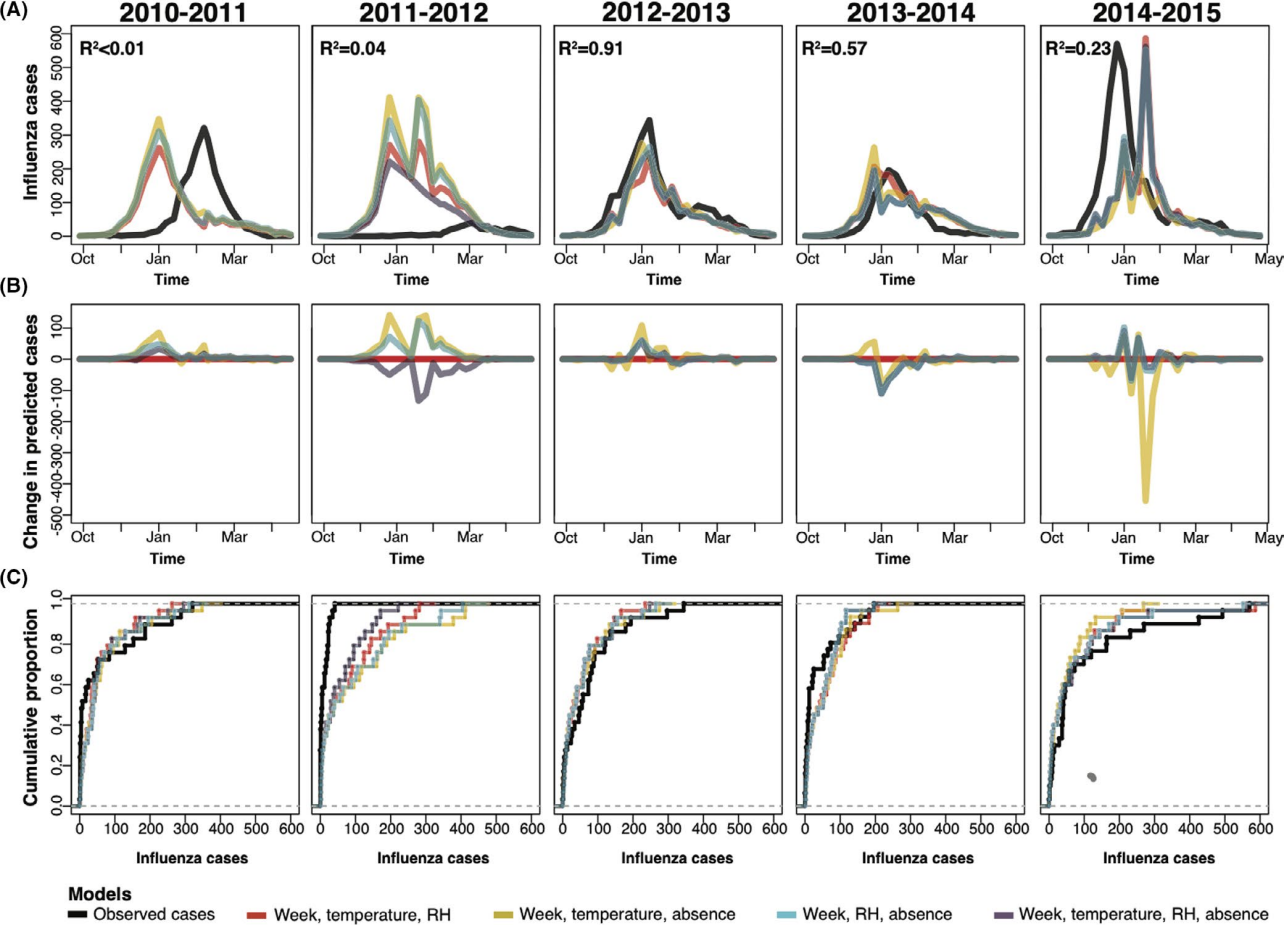
Four model predictions of confirmed influenza in Allegheny County using leave‐one‐season‐out validations for the 2010 to 2014 influenza seasons. Model predictions of four negative binomial models (calendar week, average weekly temperature, average weekly relative humidity (red), one‐week‐lagged county‐level all‐cause absences, temperature, and week model (yellow), one‐week‐lagged county‐level all‐cause absences, relative humidity, and week model (blue), and one‐week‐lagged county‐level all‐cause absences, temperature, relative humidity, and week model (purple)) using leave‐one‐season‐out validation approaches showing model predictions compared to observed virologically confirmed influenza cases (black line) in Allegheny County, Pennsylvania, USA, (A) weekly counts during each of the 2010‐2011 to 2014‐2015 influenza seasons, (B) the change in predicted cases using modeling including absences compared to a seasonal model excluding absences (red), and (C) the cumulative proportions of predicted and observed influenza cases for each season. *R* ^2^ was obtained using a linear regression, where the observed cases from the left‐out season are the dependent variable and the independent variable was predicted cases from a negative binomial model of week‐lagged county‐level all‐cause absences, relative humidity, temperature, and calendar week

For individual influenza seasons, weekly lagged country‐level absence multivariate models predicted low‐severity seasons ^26^ (ie, 2010‐2011, 2011‐2012) poorly, but predicted more moderately severe seasons (ie, 2012‐2013, 2013‐2014) with relatively high accuracy (*R* ^2^ of 0.91 and 0.57) (Figure 2A). Predicted seasonal peaks were earlier and over‐predicted during low transmission seasons (ie, 2010‐2011 and 2011‐2012), whereas during high transmission seasons (2014‐2015) had later predicted peaks, but of equal magnitude (Figure 2A,C). Compared to seasonal models, predicted cases from all‐cause absence models varied (either increased or decreased) over the five seasons (Figure 2B), with seasonal peak timing varying most (Figure 2B). Calendar week, average weekly temperature, and absence models varied the most across seasons (Figure 2B). The model containing all seasonal variables and weekly absences had the smallest changes in predicted cases. Lowest MAE models depended on the withheld validation season (Supplemental Table 5). Given the consistently low MAEs of the model including calendar week, average weekly temperature, average weekly relative humidity, and school absence, we present results from this model.

### 3.3 Influenza predictions using school‐type and grade‐specific absences

We compared the performance of different school types (elementary, middle, and high school) and grade‐specific absences in seasonal models. Elementary school models had lower relMAEs compared to middle and high school models across validations (Supplemental Table 6). Given varied model performance across school types, we also considered one‐week‐lagged grade‐specific all‐cause absences in seasonal models to assess heterogeneity in predictions by grades.

**FIGURE 3:**
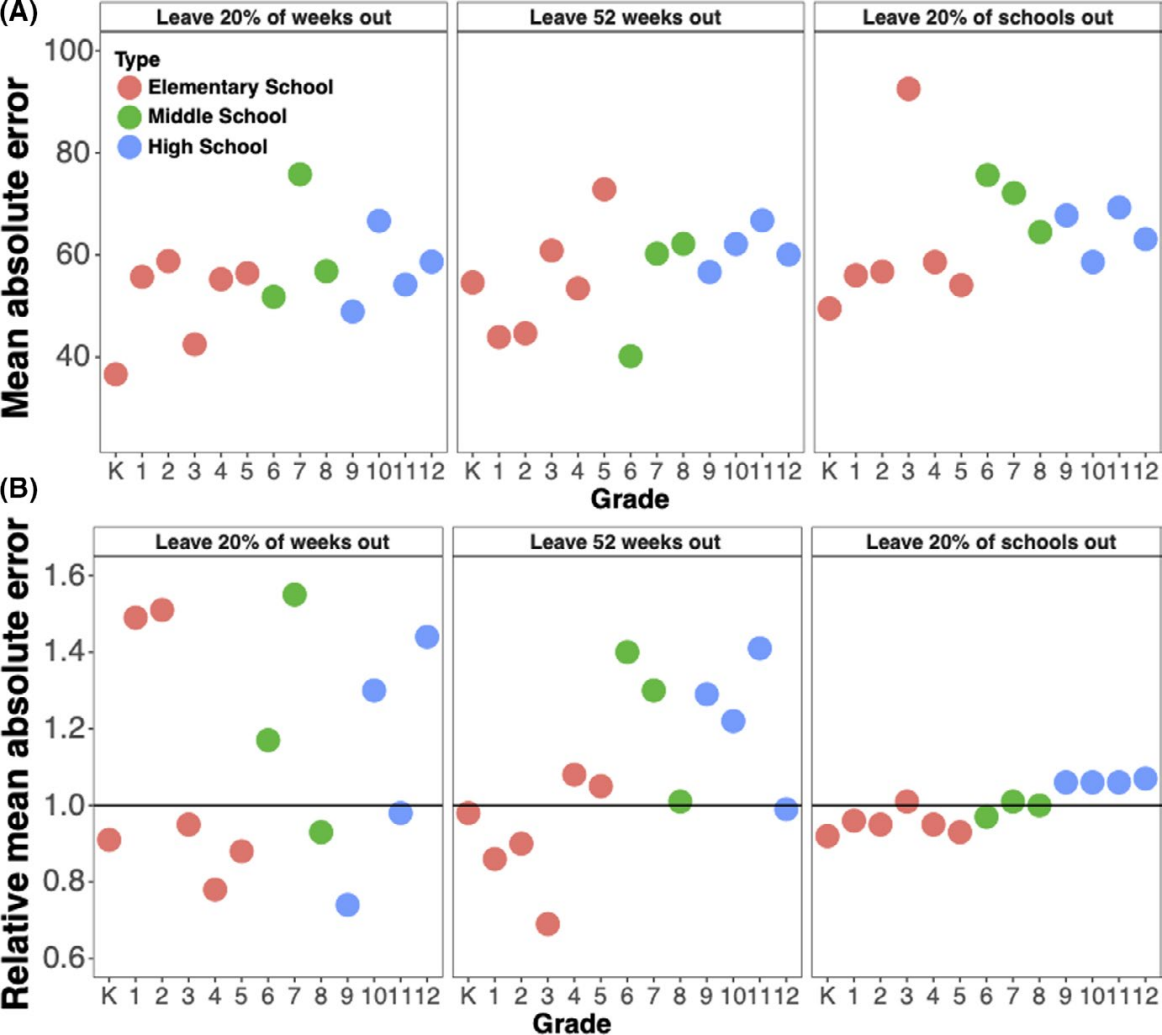
Mean and relative absolute errors for predictions using grade‐specific absence models to predict influenza in Allegheny County Pennsylvania from 2010 to 2014 influenza seasons. Mean absolute errors were estimated from univariate grade‐specific weekly absences models (A), and the relative mean absolute error compared models of grade‐specific weekly absences, week of the year, average weekly relative humidity, average weekly temperature to models of calendar week, average weekly relative humidity, and average weekly temperature (B). Colors reflect the three different school types: red is elementary school, green is middle school, and blue is high school. Solid black line refers to a relMAE of 1, where mean absolute errors of the grade‐specific absence models and models excluding absences are the same

Univariate analyses found K, 1, 2, 3,4th, and 5th grade absence models had lower MAEs than (individual) middle school and high school grade‐specific absence models, particularly in leave 20% of schools’ out validation (Figure 3A). Multivariate grade‐specific absence models also had lower MAEs relative to seasonal models across three cross‐validations (Figure 3B). We observed consistently lower relMAEs for kindergarten‐specific absences (relMAE: 0.91, 0.98. 0.92 in three validations). Overall, middle and high school grade‐specific absence models did not decrease MAEs relative to seasonal models, although 8, 9, and 10th grade models in leave 20% of weeks out and 6th grade models in leave 20% schools out had lower MAEs.

We investigated whether absenteeism can be used to create more accurate predictions of virologically confirmed influenza only in school‐aged children, and we built models of virologically confirmed influenza only in those 5‐17 years old, rather than of all ages. We found modest improvements in two of three validations when including absences compared to not using absences in models that incorporated week of year, relative humidity, and temperature. Predictions were more accurate when predicting virologically confirmed influenza in children than when predicting all ages.

### 3.4 Influenza predictions comparing all‐cause and influenza‐like illness‐specific absences from cohort data

Using school‐based cohort studies, we compared the performance of all‐cause absences to ILI‐specific absences, a better proxy for influenza infection. Because the cohorts had short time‐series (ie, one influenza season), we were unable to examine models containing all seasonal variables and to include average temperature in some models. Multivariate ILI absence models had higher *R* ^2^ estimates and lower relMAEs than all‐cause absence models in analyses using PIPP, 2012‐2013 SMART, and pooled absence data (Table 2). From the 2015 to 2016 SMART^2^ data, the all‐cause absence model had a lower relMAE (relMAE: 0.59) than the ILI‐specific model (relMAE: 2.17), but similar *R* ^2^ estimates (Table 2). Pooling across studies, the ILI‐specific absence model had a lower relMAE (relMAE:0.99) than the all‐cause absence model (relMAE: 1.02), and similar *R* ^2^ estimates (all‐cause *R* ^2^: 0.30 and ILI‐specific *R* ^2^: 0.37) (Table 2).

**TABLE 2:** All‐cause and cause‐specific absences model performance using three school‐based cohorts’ data to predict confirmed influenza cases in Allegheny County, Pennsylvania, USA during the 2007‐2008, 2012‐2013, and 2015‐2016 influenza seasons

Flu season	Cohort	Model^a^, ^b^, ^c^	In‐sample AICc (∆)	*R* ^2^	MAE	Relative MAE
2007‐2008	PIPP	Week‐only	183 (0)	0.97	8.9	1.0 (Ref.)
		All‐cause absence	215 (32)	0.44	158.6	17.8
		ILI‐specific absence	185 (2)	0.49	45.1	5.07
2012‐2013	SMART	Week‐only	161 (0)	0.93	14.4	1.0 (Ref.)
		All‐cause absence	187 (26)	0.98	11.2	0.78
		ILI‐specific absence	174 (13)	0.99	8.6	0.60
2015‐2016	SMART^2^	Week‐only	204 (0)	1.0	8.6	1.0 (Ref.)
		All‐cause absence	214 (10)	0.82	5.0	0.59
		ILI‐specific absence	206 (2)	0.84	18.6	2.17
Pooled analysis	PIPP, SMART, SMART^2^	Week‐only	664 (0)	0.35	51.6	1.0 (Ref.)
		All‐cause absence	665 (1)	0.30	52.4	1.02
		ILI‐specific absence	667 (3)	0.37	51.5	0.99

Abbreviations: ∆AICc: change in corrected Akaike's Information Criterion; ILI: influenza‐like‐illness; MAE: mean absolute error; PIPP: Pittsburgh Influenza Prevention Project; *R* ^2^: coefficient of determination; SMART: Social Mixing and Respiratory Transmission in Schools study; SMART2: Surveillance, Monitoring of Absences & Respiratory Transmission Study.

^a^ The week‐only model included only week of the year and absence models included weekly lagged absence rates from the previous week, week of the year, and average temperature.

^b^ SMART models included weekly lagged absence rates and week of the year.

^c^ Cross‐validation used leave 20% of schools out.

### 3.5 Sensitivity analyses

In sensitivity analyses, we found using absence duration did not improve model predictions. One‐day absence models and those including both one‐day and absences two days or more had lower relMAEs compared to models containing absences two days or more (Supplemental Table 9), but predictions from the three models did not substantially vary (Supplemental Figure 2). Evaluation of models including one‐week‐lagged influenza cases found little improvement of model prediction and performance when compared to seasonal models. Higher MAEs were observed for one‐week‐lagged influenza models, and one‐week‐lagged influenza and absence models but had similar *R* ^2^ estimates (Supplemental Table 8). One exception was the one‐week‐lagged influenza model from the leave 20% schools’ out validation, which had a lower relMAE (relMAE: 0.97) (Supplemental Table 8). One‐week‐lagged influenza and one‐week‐lagged influenza and kindergarten absence models performed similarly to one‐week‐lagged influenza models in three cross‐validations, except in the leave 52 weeks out validation (relMAE: 0.97).

## 4 DISCUSSION

We found including school absences in seasonal models improved community‐level confirmed influenza predictions over multiple seasons within Allegheny County. All‐school absence models subtly improved predictions, reducing MAE by 5% across multiple validations, but school‐ and grade‐specific absence models had better predictions, reflecting underlying age‐specific differences in infections. Elementary school absence (K to 5th grades) models decreased MAEs by 1%‐16% compared to 6‐12th grades, suggesting younger student absences were illness‐related and older children's absences were non‐influenza and non‐illness‐related. From school cohort data, ILI‐ and all‐cause absences performed better in single season (2007‐2008 and 2012‐2013) validations and when pooled across seasons. Elementary school, K‐5th grade‐specific all‐cause absences, and potentially ILI‐specific absences may serve surveillance indicators for the larger community.

Compared to seasonal models, those including all‐cause absences improved MAE and *R* ^2^ estimates and suggests that after accounting for seasonal factors, school absences improved influenza predictions. Our analysis is one of few using weekly all‐cause absences at various administrative levels (ie, school type and grades) to predict influenza. Whereas other studies used cause‐specific absences to detect elementary school influenza outbreaks,^6^ ours evaluated how different school and grade all‐cause absences performed as predictors. As evidenced by higher *R* ^2^ and lower relMAEs from elementary school absence models, absences from younger school‐aged children better reflect infections during the influenza season and are a proxy to the younger age groups that experience higher infections and increased susceptibility.^5^, ^25^, ^27^ In contrast, middle and high schools’ absences were noisier prediction signals, possible because older students had more non‐influenza‐related absences (consistent with the overall higher absenteeism rates observed in these schools over time). Lower relMAEs from lower individual grade (K‐5th grades) absence models from multiple validations further support our findings. Hence, elementary school absences could be useful for influenza surveillance.

ILI‐specific absences predicted influenza better than all‐cause absences when evaluating predictions from weekly all‐cause and ILI‐specific absence models (using school‐based cohort studies), based on lower MAEs and higher *R* ^2^ for specific seasons and when pooled. Other studies also found ILI‐specific absences were a proxy for influenza when evaluating vaccine impacts,^28^ suggesting ILI‐specific absences likely capture actual influenza infections. We could not conduct cause‐specific absence surveillance for more than one influenza season for each study nor could we perform school‐type and grade‐specific comparisons of all‐cause and ILI‐specific absences due to small time‐period, but these may also be important predictors of influenza incidence.

Our study has some limitations. We did not evaluate our predictions during the 2009 pandemic because our county absence data were either limited to single seasons, or available after 2009 because participating schools’ electronic absence surveillance began after 2009. Similarly, cohort studies were funded for and conducted during the 2007, 2012, and 2015 seasons; therefore, we could not assess predictions during the 2009 pandemic. In the school‐based cohort studies, not all absences were identified due to challenges contacting parents regarding absences and our studies may underestimate the number of all‐cause absences, and possibly, ILI‐specific absences. Our predictions used school‐based data from school districts within Allegheny County only; therefore, our results may not be generalizable to influenza transmission in other US counties. Additional data from other Pennsylvania counties or a representative sampling from other state counties would improve the generalizability of our predictions.

Recently, others, like those participating in the CDC FluSight Challenge—an influenza prediction competition—have used climate data, past influenza incidence, and other data streams in recent efforts. In the CDC FluSight Challenge, external research teams predict weekly influenza cases, and evaluation metrics include the mean absolute scaled error, a measure of forecast accuracy.^29^, ^30^ Our MAE decreased by 5% when using county‐level all‐cause absences models and is equivalent an additional 8 weeks of data included in a nowcast model, like those used in the FluSight Challenge. This equates to a 5% reduction in mean absolute scaled error.^31^ Our results suggest that models including lower grades’ absences may improve predictions, as seen by the 10% MAE decrease, and may improve predictions more when incorporated into ensemble models, like those used in FluSight.^30^

Our findings suggest models using absences of younger students improve predictive performance. Real‐time, day‐to‐day absence data are easy to collect, readily available in many schools, and can provide more accurate predictions than other surveillance mechanisms reliant on virologic confirmation, and susceptible to laboratory testing delays. Future studies could apply absence data to other prediction methodologies, like ensemble methods and machine‐learning algorithms, which may improve prediction accuracy and identify absence‐related patterns not considered here. We demonstrate grade‐specific all‐cause absences predict community‐level influenza one‐week forward, when influenza‐ or cause‐specific absences are unavailable and suggest elementary school or lower grade absenteeism during the influenza season can reflect influenza circulation. Using school indicators can inform influenza surveillance and control efforts, including annual vaccination; antiviral treatment or prophylaxis; and promotion of everyday preventive measures (ie, staying home when sick, respiratory hygiene, and hand hygiene) to reduce school‐ and community‐level influenza transmission.

## CONFLICT OF INTEREST

The authors declare that they have no conflicts.

## AUTHOR CONTRIBUTION

**Talia M. Quandelacy:** Conceptualization (equal); Data curation (equal); Formal analysis (equal); Writing‐original draft (equal); Writing‐review & editing (equal). **Shanta Zimmer:** Conceptualization; Funding acquisition (equal); Writing‐review & editing (equal). **Justin Lessler:** Conceptualization (equal); Supervision (equal); Writing‐review & editing (equal). **Charles VUKOTICH:** Project administration (equal); Writing‐review & editing (equal). **Rachel Bieltz:** Project administration (equal). **Kyra Grantz:** Project administration (equal); Writing‐review & editing (equal). **David Duane Galloway:** Data curation (equal); Project administration (equal). **Jonathan M Read:** Funding acquisition (equal); Writing‐review & editing (equal). **Yenlik Zheteyeva:** Project administration (equal); Writing‐review & editing (equal). **Hongjiang Gao:** Project administration (equal); Writing‐review & editing (equal). **Amra Uzicanin:** Project administration (equal); Writing‐review & editing (equal). **Derek AT Cummings:** Conceptualization; Funding acquisition (equal); Supervision (equal); Writing‐review & editing (equal).

## Supporting information

Supplementary Material

## Data Availability

The data that support the findings of this study are available from the corresponding author upon reasonable request.
