# Supplementary material for: Predicting virologically confirmed influenza using school absences in Allegheny County, Pennsylvania, USA during the 2007‐2015 influenza seasons
Source: Influenza Other Respir Viruses. 2021 Sep 3;15(6):757–66. doi: 10.1111/irv.12865 (PMC8542956; doi:10.1111/irv.12865)
Supplement: Supplementary file 1 — Supplementary Material [file IRV-15-757-s001.docx]

**Supplemental Information**

*Data*

Nine school districts provided county-level absences: Bethel Park (8 schools, N=4,300); Deer Lakes (4 schools, N=1,900); East Allegheny County (3 schools, N=1,600); Pine Richland (6 schools, N= 4,650); Pittsburgh City (78 schools, N=25,000); Propel Charter (10 schools, N=3,600); Quaker Valley (4 schools, N=2,500); South Fayette (4 schools, N=2,900); and West Jefferson Hills (5 schools, N=2,850), with 98 primary and secondary schools (K-12 grades) and 24 accelerated academies and charter schools, residing in Allegheny County.

In the school-based cohort studies 11,600 students participated. Specifically, 3,810 students from ten K-5 schools from one district participated in the PIPP study, 2,694 students from nine K-12 schools across two school districts participated in the SMART study from 2012-2013, and 4,312 students from nine K-12 schools across three school districts participated in SMART^2^ 2015-2016. During the PIPP study, 1,839 all-cause absences and 486 ILI-specific absences occurred; 2,251 all-cause absences and 1,669 ILI-specific absences occurred during the SMART study in 2012-2013; and 15,487 all-cause and 685 cause-specific absences during the SMART^2^ study in 2015-2016.

The respective average weekly all-cause and ILI-specific absence rates were 0.4 weekly absences/100 students (IQR: 0.3, 0.5) and 0.1 weekly absences/100 students (IQR: 0.0, 0.2) weekly absences/100 students in the PIPP study, 1.6 weekly absences/100 students (IQR: 1.1, 1.8) and 1.1 weekly absences/100 students (IQR: 0.7, 1.4) in the SMART study, and 2.2 weekly absences/100 students (IQR: 1.8, 2.5) and 0.10 weekly absences/100 students (IQR: 0.0, 0.12) in the SMART^2^ study.

*Absence-lag assessment*

We examined different week lags for weekly school absences rate in models of week of year, average relative humidity and average weekly temperature. Compared to a non-lagged model (i.e. the reference model), the in-sample model AICc increased for all lagged models with the exception of the two-week lagged model in the leave 20% randomly sampled schools out ($\Delta$AICc=0) (Supplemental Table 2). The highest R^2^ in three validations were from the one-week lagged model (R^2^: 0.53 for leave 20% randomly sampled data out and R^2^:0.54 for leave 20% randomly sampled schools out), and from the three-week lagged model (R^2^: 0.54 for leave 52 weeks out). The lowest mean absolute errors relative to the non-lagged model were the one-week lagged model for two validations (relMAE: 0.84, 1.0 for leave 20% randomly sampled data out and leave 52 randomly sampled weeks out), and the three-week lagged model for leave 20% randomly sampled data out (relMAE: 0.97). Similar trends were observed for leave-one-season out validation (Supplemental Table 3). While the two-week lagged model had similar $\Delta$AICc and R^2^ values, and the three-week lagged model occasionally had a lower relative MAE, we used the one-week lagged model, given similarity in model performance and based on the infectious period of influenza spread within one week.

*Model comparisons*

When performing leave-one-season-out cross-validation, predictions depended on the influenza season withheld. Similarly, the model with the lowest relative mean absolute error varied based on season withheld, though the model including week of year, average temperature, average weekly relative humidity, and one-week lagged absences had lower mean absolute errors for the 2012-2013 (relMAE=0.86) and 2014-2015 season (relMAE=0.96) (Supplemental Table 4). For other seasons, the lowest mean absolute errors were from models containing week of year, average weekly temperature, and absences or containing week of year, average weekly relative humidity, and absences.

*School-level absence predictions*

One-week lagged absence rates from different school types improved influenza predictions over using county-level absences. Specifically, elementary school absences improved performance compared to middle and high school absences across three cross-validations (Supplemental Table 5) based on R2 and relative mean absolute errors. The mean absolute errors of elementary school absence models were lower relative to the reference model (i.e. week of year, average weekly relative humidity, and average weekly temperature) across the three validations (relMAE: 0.94, 0.98, 0.99), and also had lower relative mean absolute errors compared to the middle (relMAE: 0.96, 1.0, 1.02 for leave 20% randomly sampled data out, leave 52 randomly sampled weeks out, and leave 20% of randomly sampled schools out, respectively) and high school absence models (relMAE: 0.99, 1.03, 1.02 for leave 20% randomly sampled data out, leave 52 randomly sampled weeks out, and leave 20% of randomly sampled schools out, respectively) (Supplemental Table 6). Elementary school absences performed similarly to or worse than middle and high schools in leave-one-season-out validation (Supplemental Table 7).

**Supplemental Table 1. Characteristics of confirmed influenza cases, student population, and reported absences during the 2007-2008, and 2010-2015 influenza seasons in Allegheny County, Pennsylvania, USA**

| Season | 2007-2008 | 2010-2011 | 2011-2012 | 2012-2013 | 2013-2014 | 2014-2015 | 2015-2016 | Total |
| --- | --- | --- | --- | --- | --- | --- | --- | --- |
| Predominant circulating strain | A/H1N1**^†^** | A/H3N2 | A/H3N2 | A/H3N2 | A/H1N1 | A/H3N2 | A/H1N1 |  |
| Overall influenza cases | 1.267 | 1,648 | 301 | 2,445 | 1,312 | 3,150 | 1,823 | 11,946 |
| Influenza A cases | 699 | 1,091 | 262 | 1,941 | 1,209 | 2,871 | 1,277 | 9,350 |
| A/H3N2 cases | 21 | 31 | N/A | 274 | 6 | 1,046 | 19 | 1.397 |
| A/H1N1 cases | 3 | 188 | 11 | 14 | 445 | 16 | 439 | 1,116 |
| B cases | 554 | 508 | 15 | 480 | 81 | 266 | 539 | 2,453 |
| County school population and absences | | | | | | | | |
| Number of students | N/A | 33,614* | 46,695 | 46,234 | 46,076 | 45,562 | N/A |  |
| Number of All-cause absences | N/A | 405,182 | 385,188 | 493,137 | 463,663 | 437,050 | N/A | 2,184,220 |
| School-based cohort studies | | | | | | | | |
|  | **PIPP** |  |  | **SMART** |  |  | **SMART^2^** |  |
| Number of students | 3,810 | N/A | N/A | 2,694 | N/A | N/A | 5,156 | 11,660 |
| Number of All-cause absences | 1,839 | N/A | N/A | 2,251 | N/A | N/A | 16,039 | 20,129 |
| Number of ILI-specific absences | 486 | N/A | N/A | 1,839 | N/A | N/A | 687 | 3,012 |
| Influenza season is defined as the period between the 40th week of one year and the 20th week of the subsequent calendar year. Subtype estimates were provided for isolates where such information was available. *County-level data for 2010-2011 season were not available for Bethel, Deer Lakes, and Pine Richland. N/A refers to data not available during years out of the cohort study periods. † H1N1 after 2009 refers to A/H1N1pdm09. Abbreviations: H: hemagglutinin; N: neuraminidase; A: influenza type A; B: influenza type B; ILI: Influenza-like illness; NS: not subtyped; PIPP: Pittsburgh Influenza Prevention Project; SMART: Social Mixing and Respiratory Transmission in Schools study; SMART^2^: Surveillance, Monitoring of Absences & Respiratory Transmission study. | | | | | | | | |

**Supplemental Table 2. Fits and prediction measures comparing seasonal models to varying week-lags of all-county all-cause absence rates**

| Model validation | Leave 20% randomly sampled data out (n=110) | | | | Leave 52 randomly sampled weeks out (n=87) | | | | Leave 20% of randomly sampled schools out (n=157) | | | |
| --- | --- | --- | --- | --- | --- | --- | --- | --- | --- | --- | --- | --- |
| Model | **∆AICc** | **R^2^** | **MAE** | **relMAE** | **∆AICc** | **R^2^** | **MAE** | **relMAE** | **∆AICc** | **R^2^** | **MAE** | **relMAE** |
| No lag (Ref.) | 0 | 0.13 | 43 | 1.0 | 0 | 0.21 | 37 | 1.0 | 0 | 0.50 | 36 | 1.0 |
| One-week lag | 6 | 0.53 | 36 | 0.84 | 4 | 0.45 | 37 | 1.0 | 1 | 0.52 | 36 | 1.0 |
| Two-week lag | 5 | 0.48 | 37 | 0.86 | 2 | 0.38 | 40 | 1.1 | 0 | 0.54 | 36 | 1.0 |
| Three-week lag | 6 | 0.48 | 38 | 0.88 | 4 | 0.41 | 38 | 1.1 | 1 | 0.56 | 35 | 0.97 |
| Each negative binomial model included weekly county-level all-cause absence rates at various lags, week of year, average weekly temperature, & average relative humidity. We used generalized additive models to estimate the effective degrees of freedom for seasonal variable. Change in AICc compared the AICc of the 1-,2-,3-week lagged models to the non-lagged model (reference model). R^2^ is the correlation between the observed and predicted influenza cases and was estimated using linear regression with the observed cases as the dependent variable and the predicted cases as the independent variable. Mean absolute error is the difference in predicted cases and out-of-sample observed cases divided by the number of out-of-sample weeks. the relative mean absolute error compares the MAE of the varying week lagged models to the MAE non-lagged model. Abbreviations: n: number of in-sample observations; AICc, Akaike’s Information Criterion; relMAE, relative mean absolute error; R^2^, correlation; Ref., Reference | | | | | | | | | | | | |

**Supplemental Table 3. Fit and performance of models containing varying week-lags of weekly school absences to predict influenza cases in Allegheny County, PA using leave one season out validation**

| Season left out | 2010-2011 (n=109) | | 2011-2012 (n=113) | | 2012-2013 (n=110) | | 2013-2014 (n=109) | | 2014-2015 (n=111) | | |
| --- | --- | --- | --- | --- | --- | --- | --- | --- | --- | --- | --- |
| **Model** | **∆AICc** | **relMAE** | **∆AICc** | **relMAE** | **∆AICc** | **relMAE** | **∆AICc** | **relMAE** | **∆AICc** | **relMAE** | |
| **No lag (Ref.)** | 0 | 1.0 | 0 | 1.0 | 0 | 1.0 | 0 | 1.0 | 0 | 1.0 | |
| **One-week lag** | 1 | 1.0 | -2 | 1.4 | 2 | 1.2 | -2 | 1.4 | 0 | 1.0 | |
| **Two-week lag** | -1 | 1.0 | 1 | 1.1 | 1 | 1.2 | -2 | 1.6 | 0 | 1.1 | |
| **Three-week lag** | 1 | 0.99 | -1 | 1.1 | 2 | 1.3 | 2 | 1.3 | 1 | 0.99 | |
| Each negative binomial model included weekly absence rates at various week lags, and week of the year, average weekly relative humidity and average weekly temperature modeled splines. Change in AICc compared the in-sample AICcs of lagged models to those of the non-lagged model (reference model). R^2^ was obtained by linearly regressing the observed cases against the predicted cases using the out-of-sample data. Mean absolute error is the mean of the absolute prediction error over the time series. Abbreviations: n: number of in-sample observations; ∆AICc: Akaike’s Information Criterion, corrected for small sample sizes; MAE: mean absolute error; relMAE: relative mean absolute error; R^2^: coefficient of determination, Ref: Reference | | | | | | | | | | |  |

**Supplemental Table 4. Performance of negative binomial models excluding and including weekly all-cause county-level school absence rates across different validations to predict virologically confirmed influenza cases in Allegheny County, Pennsylvania, USA, during 2010-2011 to 2014-2015 influenza seasons**

| Model validation  (in-sample obs.) | | Leave 20% randomly sampled data out (n=124) | | Leave 52 randomly sampled weeks out (n=106) | | Leave 20% randomly sampled schools out (n=159) | |
| --- | --- | --- | --- | --- | --- | --- | --- |
| Model | **Variables** | **MAE** | **relMAE** | **MAE** | **relMAE** | **MAE** | **relMAE** |
| 1 (Ref.) | Week of year, avg. weekly temp., average weekly RH | 39 | 1.0 | 43 | 1.0 | 37 | 1.0 |
| 2 | Week of year, Avg. weekly temp., weekly abs. rates | 38 | 0.97 | 45 | 1.05 | 37 | 1.0 |
| 3 | Week of year, Avg. weekly RH, Weekly abs. rates | 48 | 1.23 | 54 | 1.24 | 47 | 1.27 |
| 4 | Week of year, Avg. weekly temp., Avg. weekly RH, Weekly abs. rates | 37 | 0.95 | 43 | 1.0 | 35 | 0.95 |
| Each negative binomial model included weekly county-level all-cause absence rates at various lags, week of year, average weekly temperature, & average relative humidity. We used generalized additive models to estimate the effective degrees of freedom for seasonal variable. Mean absolute error is the difference in predicted cases and out-of-sample observed cases divided by the number of out-of-sample weeks. the relative mean absolute error compares the MAE of the models including absences to the MAE of the seasonal variable only model (i.e. Model 1). Abbreviations: n: number of in-sample observations; MAE, mean absolute error; relMAE, relative mean absolute error; Ref., Reference | | | | | | | |

**Supplemental Table 5. Model performance using leave-one-season-out cross validation of seasonal variables including and excluding county-level all-cause absence rates to predict county-level confirmed influenza**

| Season left-out (n=in-sample obs.) | | 2010-2011 (n=127) | | | 2011-2012 (n=126) | | | 2012-2013 (n=126) | | | 2013-2014 (n=124) | | | 2014-2015 (n=121) | | |  |
| --- | --- | --- | --- | --- | --- | --- | --- | --- | --- | --- | --- | --- | --- | --- | --- | --- | --- |
| Model | **Variables** | **AICc** | **MAE** | **relMAE** | **AICc** | **MAE** | **relMAE** | **AICc** | **MAE** | **relMAE** | **AICc** | **MAE** | **relMAE** | **AICc** | **MAE** | **relMAE** |  |
| 1 (Ref.) | Week of year, avg. weekly temp., avg. weekly RH | 0 | 73 | 1.0 | 0 | 74 | 1.0 | 0 | 28 | 1.0 | 0 | 26 | 1.0 | 0 | 56 | 1.0 |  |
| 2 | Week of year, avg. weekly temp., weekly abs. rates | 2 | 77 | 1.05 | -2 | 91 | 1.23 | -4 | 24 | 0.86 | -4 | 29 | 1.12 | -1 | 55 | 0.98 |  |
| 3 | Week of year, avg. weekly RH, weekly abs. rates | -1 | 80 | 1.10 | 12 | 38 | 0.5 | -1 | 42 | 1.50 | -1 | 40 | 1.54 | -1 | 70 | 1.25 |  |
| 4 | Week of year, avg. weekly temp., avg. weekly RH, weekly abs. rates | 1 | 78 | 1.07 | 1 | 91 | 1.23 | 1 | 24 | 0.86 | 1 | 29 | 1.12 | 1 | 54 | 0.96 |  |
| Each model used negative binomial regression and used generalized additive models to estimate degrees of freedom for non-linear variables. Weekly absence rates were county-level weekly all-cause absence rates lagged one-week. Reference models were the seasonal variable model for both changes in AIC and relMAE comparisons. Mean absolute error was estimated as the prediction error averaged over the number of weeks in the out of sample validation set. Relative MAE=1 refers to equivalent mean absolute errors of the two models. Abbreviations: abs., absence; avg, average; temp, temperature; RH, relative humidity; AICc, Aikaike’s information criterion corrected for small sample sizes; MAE, mean absolute error; relMAE, relative mean absolute error | | | | | | | | | | | | | | | | | |

| Model validation (n=in-sample obs) | Leave 20% randomly sampled data out (n=127) | | | | Leave 52 randomly sampled weeks out (n=107) | | | | Leave 20% randomly sampled schools out (n=159) | | | |
| --- | --- | --- | --- | --- | --- | --- | --- | --- | --- | --- | --- | --- |
| Model | **AICc** | **R^2^** | **MAE** | **relMAE** | **AICc** | **R^2^** | **MAE** | **relMAE** | **AICc** | **R^2^** | **MAE** | **relMAE** |
| Elementary school | 1023 | 0.12 | 31 | 0.94 | 854 | 0.27 | 33 | 0.98 | 1297 | 0.52 | 34 | 0.99 |
| Middle school | 1022 | 0.02 | 34 | 0.96 | 842 | 0.43 | 48 | 1.0 | 1278 | 0.51 | 35 | 1.02 |
| High school | 1023 | 0.02 | 35 | 0.99 | 855 | 0.13 | 35 | 1.03 | 1279 | 0.52 | 35 | 1.02 |
| Each negative binomial model included one-week lagged all-cause absence rates, week of year, average relative humidity, and average weekly temperature. R^2^ was estimated using linear regression with the observed cases as the dependent variable and the predicted cases as the independent variable. Mean absolute error is the mean of the absolute difference in predicted cases and observed cases. The relative mean absolute error compared the MAE from a multivariate school absence model to a model containing week of year, average weekly relative humidity and average weekly temperature. Abbreviations: AICc, corrected Akaike’s Information Criterion for small sample sizes; MAE, mean absolute error; relMAE, relative mean absolute error, R^2^, correlation coefficient. | | | | | | | | | | | | |

**Supplemental Table 6. Fit and prediction measures of seasonal variable models including elementary, middle school or high school weekly all-cause absence rates to predict confirmed influenza in Allegheny County, Pennsylvania during 2010-2015 influenza seasons**

**Supplemental Table 7. Fit and prediction measures of seasonal variable models including elementary, middle school or high school weekly all-cause absence rates using leave-one-season-out validation to predict confirmed influenza in Allegheny County, Pennsylvania during 2010-2015 influenza seasons**

| Model validation (n=in-sample obs) | 2010-2011  (n=120) | | | 2011-2012  (n=118) | | | 2012-2013  (n=119) | | | 2013-2014  (n=118) | | | 2014-2015  (n=117) | | |
| --- | --- | --- | --- | --- | --- | --- | --- | --- | --- | --- | --- | --- | --- | --- | --- |
| Model | **AICc** | **MAE** | **relMAE** | **AICc** | **MAE** | **relMAE** | **AICc** | **MAE** | **relMAE** | **AICc** | **MAE** | **relMAE** | **AICc** | **MAE** | **relMAE** |
| Elementary school | 1011 | 71 | 1.07 | 1007 | 84 | 1.13 | 997 | 22 | 0.92 | 1028 | 30 | 1.28 | 946 | 86 | 1.12 |
| Middle school | 1012 | 68 | 1.01 | 1005 | 77 | 1.03 | 999 | 20 | 0.86 | 1035 | 24 | 1.08 | 946 | 79 | 1.0 |
| High school | 1013 | 67 | 1.00 | 1003 | 93 | 1.25 | 1001 | 22 | 0.92 | 1038 | 22 | 1.02 | 945 | 81 | 1.03 |
| Each negative binomial model included one-week lagged all-cause absence rates, week of year, average relative humidity, and average weekly temperature. R^2^ was estimated using linear regression with the observed cases as the dependent variable and the predicted cases as the independent variable. Mean absolute error is the mean of the absolute difference in predicted cases and observed cases. The relative mean absolute error compared the MAE from a multivariate school absence model to a model containing week of year, average weekly relative humidity and average weekly temperature. Abbreviations: AICc, corrected Akaike’s Information Criterion for small sample sizes; MAE, mean absolute error; relMAE, relative mean absolute error, R^2^, correlation coefficient. | | | | | | | | | | | | | | | |

**Supplemental Table 8. Impact of one-week lagged influenza cases, included in models of county-level and kindergarten weekly absence rates from the previous week, on model fits using different cross-validations**

**Supplemental Table 9. Impact of all-cause county-level and kindergarten-specific absences one-day in length or two-days and longer on model fits**

**Supplemental Figure 1. Weekly influenza incidence rates per 10,000, student absence rates per 10 students, average temperature, and relative humidity in Allegheny County, PA, during the 2010-2014 influenza seasons.**

**Supplemental Figure 2. Predictions of weekly reported virologically confirmed cases from 2010 to 2015 using absences of one day and two days or longer (top panel), one-day-long absences (middle panel), and absences two days or longer (bottom panel) in models of week of the year and average temperature, leaving 20% of data out.** Black lines represent observed weekly reported virologically confirmed influenza cases. Red lines represent weekly predicted influenza cases and shading represents 95% uncertainty bounds. Duration of absence was provided by six school districts providing daily student absence data.
